# Sustained impairment of human cytomegalovirus (HCMV)-specific CD4^+ ^and CD8^+ ^T cell response is responsible for recurrent episodes of disseminated HCMV infection in a D^+^R^- ^hand transplant recipient

**DOI:** 10.1186/1757-1626-1-155

**Published:** 2008-09-17

**Authors:** Fausto Baldanti, Giovanna Lucchini, Daniele Lilleri, Marco Lanzetta

**Affiliations:** 1 Servizio di Virologia, Fondazione IRCCS Policlinico San Matteo, Pavia, Italy; 2 Laboratori Sperimentali di Ricerca, Area Infettivologica, Fondazione IRCCS Policlinico San Matteo, Pavia, Italy; 3 Istituto Italiano di Chirurgia della Mano e Microchirurgia, Monza, Milano, Italy

## Abstract

Human cytomegalovirus (HCMV) infection is the major viral complication in solid organ transplant recipients. Seronegative recipents (R^-^) of organs from seropositive donors (D^+^) appear to be at higher risk of developing symptomatic HCMV infection. To what extent systemic life-threatening complications can be risked for non-life-saving transplant procedures? A case report describing successful treatment of repeated episodes of active HCMV infection in a D^+^R^- ^hand recipient in the absence of HCMV-specific T-cell immunity is presented. In the attempt to save both the patient and the transplanted hand, a preemptive treatment strategy was adopted with the aim to boost the constitution of the virus-specific T-cell immune response and simultaneously avoid onset of disease. Careful monitoring of HCMV load in blood and HCMV-specific T-cell immunity guided administration of repeated courses of antiviral treatment and avoided emergence of HCMV-related symptoms. Following establishment of HCMV-specific CD4^+ ^and CD8^+ ^T-cell response, preemptive treatment was no longer required due to sustained HCMV disappearance from blood. The patient is now well, and his hand too. In conclusion, evaluation of virus-specific T-cell immunity is of crucial importance in D^+^R^- ^transplant recipients and careful monitoring of HCMV-specific T cell mediated response should always parallel monitoring of HCMV load in transplant recipients.

## Introduction

Since the first successful hand transplantation in Lyon in 1998, 38 hands (18 unilateral and 10 bilateral) and two digits have been transplanted in 30 individuals in different countries [1]. Notwhistanding the value of such experience pioneering composite tissue transplantation and allowing to explore other transplant frontiers such as face and limb transplantation, clinicians continue to debate about the risk acceptability of such procedures [2]. Indeed, the functional and psychologic benefits of hand transplantation may be counterbalanced by the risk of potentially severe systemic complications due to immune suppression. In some instance, the clinicians may face the difficult decision of amputating a successfully transplanted hand in order to interrupt the immunosuppressive treatment in the attempt to solve a life threatening opportunistic infection. One of the most challenging viral infections in transplant recipients is human cytomegalovirus (HCMV) infection, namely in HCMV-seronegative recipients (R^-^) of grafts from HCMV-seropositive donors (D^+^) [3].

The case of a D^+^R^- ^hand transplant recipient with recurrent episodes of disseminated HCMV infection associated with impaired HCMV-specific CD4^+ ^and CD8^+ ^T cell response is described.

## Case presentation

In October 2002, a 33 year-old HCMV-seronegative caucasic male who had lost his dominant right hand at the wrist level in a car crash 10 years before was submitted to hand transplantation from a HCMV-seropositve donor. Immune suppressive treatment included tacrolimus, mycophenolate mofetil and steroids. Kinetics of HCMV infection was monitored by quantitative determination of antigenemia and DNAemia [4]. Antigenemia consists of quantitative determination of HCMV pp65-positive/2 × 10^5 ^leukocytes examined in a cytospin preparation [4]. DNAemia consists of quantitation of HCMV genome by PCR in blood and was expressed as HCMV DNA copy number/10 uL whole blood [4].

HCMV antiviral prophylaxis with i.v. ganciclovir (5 mg/Kg/die) was administered for 30 days starting fom day 2 after transplantation. Afterwards, a preemptive antiviral treatment approach was adopted. In detail, antiviral drug were administered in the absence of overt clinical symptoms when in the presence of active HCMV infection as documented by positive antigenemia values [4].

**Figure 1:**
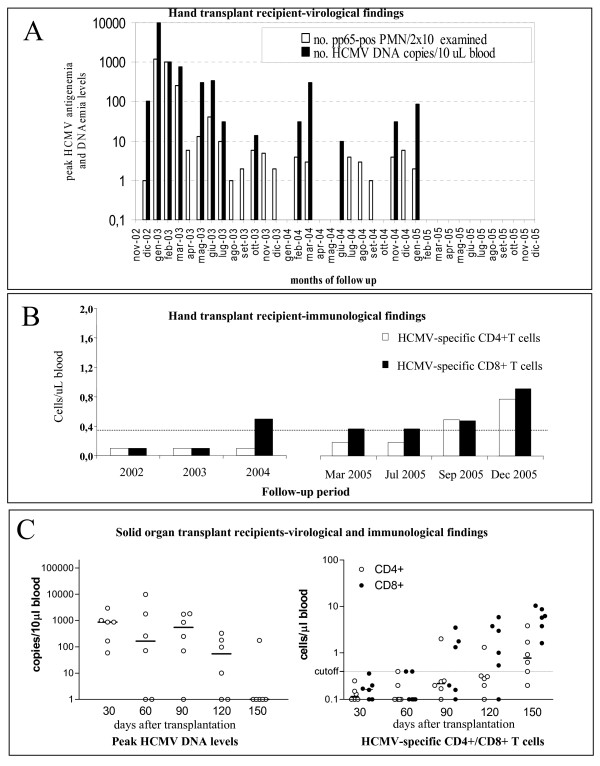
**(A) Monthly peak HCMV antigenemia and DNAemia levels in a D+R- hand transplant recipient. **(B) HCMV-specific CD4+ and CD8+ T cell counts in the same patient. (C) Monthly HCMV peak DNAemia levels and HCMV-specific CD4+ and CD8+ median T-cell counts in 6 D+R- solid organ transplant recipients undergoing primary HCMV infection and mounting an efficient T-cell immune response. The cut-off value for HCMV-specific T-cell reponse was fixed at 0.4 INFγ producing CD4+ and CD8+ T-cells/uL [10].

On day 42, a severe acute rejection episode required antithymocyte globulin administration to be controlled. Soon thereafter, HCMV antigenemia level started increasing and by day 85 reached 1200 pp65-positive/2 × 10^5 ^leukocytes examined, in the absence of overt HCMV disease. Administration of foscarnet, cidofovir and anti-CMV hyperimmune globulin was successful in controlling HCMV infection. Preemptive treatment with valgancyclovir was then scheduled to initiate at the first confirmed positive antigenemia result in order to prevent HCMV disease. During the following 3 years, antigenemia and DNAemia were found to be repeatedly positive (Figure 1A), thus prompting administration of several courses of antiviral treatment. The prolonged and repeated episodes of HCMV disseminated infection in the absence of other opportunistic infections suggested a potential selective impairment of HCMV-specific T-cell mediated immune response. The hypothesis was verified by investigating HCMV-specific CD4^+ ^and CD8^+ ^T-cell responses by a newly developed assay [5]. In detail, after in vitro differentiation from peripheral blood mononuclear cells (PBMC) separated by Lymphoprep™ (Axis-Shield PoC AS, Oslo, Norway) density gradient centrifugation, immature dendritic cells (DC) were infected for 24 h with an endotheliotropic strain of HCMV. As a control, DC were mock-infected with the clarified medium of uninfected endothelial cell cultures. HCMV-infected or mock-infected DC were cocultured overnight with thawed autologous PBMC or the non-adhering fraction of PBMC in the presence of brefeldin A (Sigma, St. Louis, MO). Then, PBMC were tested for intracellular interferon-γ(IFNγ) production by a cytokine flow cytometry assay. The frequency of CD4^+ ^and CD8^bright ^T cells producing IFNγ in response to HCMV stimuli were calculated by subtracting the value of the sample incubated with mock-infected culture medium or control antigen (consistently < 0.05%) from the test value. Absolute CD3^+^CD4^+ ^and CD3^+^CD8^+ ^T cell counts were determined on heparin-treated peripheral blood samples by a direct immunofluorescent flow cytometry method (Epics-XL, Beckman Coulter Inc.). To determine the total number of HCMV-specific CD4^+ ^and CD8^+ ^T cells, the percentages of HCMV-specific T cells positive for IFNγ were multiplied by the relevant absolute CD4^+ ^and CD8^+ ^T cell count. A cut-off value of 0.4 IFNγ- positive CD4^+ ^and CD8^+ ^T cells/μl indicated presence of HCMV-specific CD4^+ ^and CD8^+ ^T-cell immunity [6].

Due to unavailability of other D^+^R^- ^composite tissue recipients as controls, results of T-cell immunity were compared with those of some D^+^R^- ^solid organ transplant recipients (SOTR) with primary HCMV infection. Immune suppressive regimen included cyclosporine-A, mycophenolate mofetil and steroids in 1 patient, tacrolimus, mycophenolate mofetil and steroids in 2 other patients, and cyclosporina-A, azathioprine, and steroids in the remaining 2 patients.

The hand transplanted patient showed absolute CD4^+ ^T cell counts consistently < 100 for over 3 years after transplantation. HCMV-specific CD8^+ ^T cell response was below the cut-off value till 21 months after transplantation, and HCMV-specific CD4^+ ^T cell response was impaired till 35 months after transplantation (Figure 1B). Coincidental with the lack of HCMV-specific CD4^+ ^and CD8^+ ^T cell response was the consistent positivity of viral markers in blood prompting antiviral treatment, while recovery of HCMV-specific CD4^+ ^and CD8^+ ^T-cell reponses was associated with spontaneous resolution of viral infection (Figure 1A and 1B). To date, antigenemia and DNAemia remain negative in the absence of antiviral treatment. As shown in Figure 1C, control D^+^R^- ^SOTR patients developing an effective T-cell response showed HCMV-specific CD8^+ ^and CD4^+ ^T-cell levels above the cut-off value by month 3 and 4 after transplantation, respectively. In parallel with the progressive increase of the HCMV-specific T-cell response, the progressive decrease of peak HCMV DNA levels in blood was observed. As a consequence, the number of HCMV infection episodes requiring preemptive treatment in control SOTR progressively decreased.

D^+^R^- ^serostatus represents a major risk factor for severe HCMV infections in SOTR [3]. In addition, only D^+^R^- ^patients have repeatedly been reported to be at risk for development of drug-resistant HCMV infections among SOTR [4,7]. However, in the great majority of D^+^R^- ^SOTR, HCMV primary infection can be successfully controlled by antiviral treatment. In addition, immunosuppressive regimens do not avoid in the majority of SOTR development of HCMV-specific T-cell response of the same amplitude as in immunocompetent individuals [8]. On the other hand, a few reports associated the emergence of drug-resistant HCMV strains in SOTR following prolonged antiviral drug administration with the sustained impairment of HCMV-specific T-cell response [4,9,10]. Thus, the lack of HCMV-specific immunity in individual SOTR appears to play a major role in the pathogenesis of severe HCMV infections in transplanted patients.

Hand transplantation is not a life-saving procedure and it could be argued that it should be avoided in seronegative recipients since development of HCMV-specific immune response might be impaired in individual patients undergoing primary HCMV infection. In these cases, the antiviral treatment alone would be expected to be unable to control the repeated episodes of active HCMV infection with the potential risk of major complications such as development of overt disease or emergence of drug-resistant HCMV strains. For 3 years our patient was lacking HCMV-specific T cell immunity. This delay in mounting an efficient anti-HCMV cellular response is likely to be the major responsible for the recurrent episodes of disseminated HCMV infection. However, major complications were avoided in our patient by adoption of a tight virologic monitoring and repeated preemptive treatment courses. As a conclusion, a careful monitoring of HCMV-specific T-cell mediated immune response should parallel monitoring of HCMV load also in composite tissue transplant recipient.

## Abbreviations

HCMV: human cytomegalovirus; SOTR: solid organs transplant recipients; PCR: polymerase chain reaction; PBMC: peripheral blood mononuclear cells; DC: dendritic cells; IFNγ: interferonγ.

## Consent

Written informed consent was obtained from the patient for publication of this case report and accompanying images. A copy of the written consent is available for review by the Editor-in-Chief of this journal.

## Competing interests

The authors declare that they have no competing interests.

## Authors' contributions

FB and DL virologic and immunologic monitoring; ML hand transplantation and clinical management GL responsible for clinical follow-up. FB and ML are major contributors in data analysis and in writing the manuscript. All authors read and approved the final manuscript.
